# Outcomes Among Patients With Colon Cancer Living in Neighborhoods With Persistent Poverty

**DOI:** 10.1001/jamanetworkopen.2025.51212

**Published:** 2026-01-09

**Authors:** Leon Naar, Alexa L. Pohl, Arden M. Morris, Aaron J. Dawes

**Affiliations:** 1 Section of Colon & Rectal Surgery, Department of Surgery, Stanford University School of Medicine, Stanford, California; 2 Stanford-Surgical Policy Improvement Research and Education Center, Stanford University School of Medicine, Stanford, California

## Abstract

**Importance:**

Patients with cancer living in persistent poverty (PP) are at risk for worse oncologic outcomes. Existing welfare interventions typically focus on current poverty and may not benefit patients in PP if the underlying mechanisms are unique; thus, modifiable targets are needed to inform future policy efforts.

**Objectives:**

To compare clinical outcomes for patients diagnosed with colon cancer based on the share of census tracts per zip code that were in PP at the time of diagnosis and to explore 2 potential mechanisms connecting PP and disease-specific mortality.

**Design, Setting, and Participants:**

This retrospective cohort study using data from a statewide cancer registry included all patients diagnosed with colon cancer in California from 2017 to 2020. Patients with multiple malignant tumors and patients diagnosed based on only their death certificate were excluded. Data were analyzed from February 2024 to February 2025.

**Exposure:**

Proportion of census tracts per zip code designated as being in PP at the time of diagnosis (0, 0.01-0.25, 0.26-0.50, and >0.50).

**Main Outcomes and Measures:**

The primary outcome was disease-specific mortality. Secondary outcomes included overall mortality, stage at diagnosis, and receipt of guideline-concordant care. Fine-Gray competing risk survival models were used to calculate risk-adjusted mortality and to evaluate the relative contribution of access to care and quality of care as potential mediators of the association between PP and disease-specific mortality. Charlson comorbidity indices ranged from 0 to 14, with higher values indicating higher comorbidity.

**Results:**

In total, 20 015 patients (mean [SD] age at diagnosis, 65.9 [14.0] years; 51.3% male) met inclusion criteria, and the majority (66.3%) resided in zip codes with no PP. Patients living in areas with higher PP ratios were younger (eg, mean [SD] age at diagnosis, 64.3 [14.1] years for >50% PP vs 66.3 [14.1] years for no PP), more likely to identify as Hispanic (eg, 45.5% for >50% PP vs 19.2% for no PP) or non-Hispanic Black (eg, 15.7% for >50% PP vs 4.9% for no PP), and had higher Charlson comorbidity indices (eg, mean [SD] score, 1.3 [1.8] for >50% PP vs 1.2 [1.7] for no PP). After adjustment for demographic and clinical variables, higher shares of PP were associated with higher rates of disease-specific mortality: hazard ratios, 1.20 (95% CI, 1.07-1.36) and 1.19 (95% CI, 1.01-1.42) for PP ratios 0.26-0.50 and higher than 0.50, respectively. Health care practitioner density did not appear to mediate this association. However, adjusting for the receipt of guideline-concordant care affected both the magnitude and the statistical significance of the model, suggesting potential mediation.

**Conclusions and Relevance:**

In this cohort study, living in PP was associated with disease-specific mortality among patients diagnosed with colon cancer. The Persistent Poverty Initiative presents a unique opportunity to improve our understanding of PP and to support efforts to extend treatment to all US residents with cancer.

## Key Points

**Question:**

Is living in an area with persistent poverty associated with survival among patients with colon cancer?

**Findings:**

In this retrospective cohort study of 20 015 California residents diagnosed with colon cancer between 2017 and 2020, living in an area with a higher density of persistent poverty was associated with higher overall mortality and cancer-specific mortality rates. After adjusting for receipt of guideline-concordant care, the association was no longer apparent.

**Meaning:**

Findings suggest that increasing the receipt of guideline-concordant care may improve oncologic outcomes for patients with colon cancer living in areas with persistent poverty.

## Teaser

This cohort study examines clinical outcomes for patients diagnosed with colon cancer based on the share of census tracts per zip code that were in persistent poverty at the time of diagnosis and explores the relative contributions of access to care and quality of care as potential mechanisms mediating any association between persistent poverty and disease-specific mortality.

## Introduction

Social determinants of health (SDoH) continue to affect access to health care, quality of care, and clinical outcomes for US residents with cancer. Patients with cancer experiencing poverty, food insecurity, unemployment, or the effects of racially motivated housing policies are less likely to be diagnosed at an early stage, when cure is more likely, and less likely to receive recommended cancer therapies.^1,2,3,4,5,6^ Among the most alarming features of these disparities is that many appear to be modifiable, if the underlying mechanisms can be identified and addressed through targeted interventions.^7^

The US Department of Agriculture’s Economic Research Service first identified chronic exposure to poverty for its unique effects on health and human development in 1985.^8^ Since then, persistent poverty (PP)—defined as a geographic area in which more than 20% of the population has been living below the federal poverty line for 4 consecutive US censuses—has been associated with lower levels of educational attainment, higher levels of unemployment, and higher levels of both housing and food insecurity.^9^ Exposure to PP has also been associated with a higher incidence of multiple types of cancer and higher rates of cancer-specific mortality.^7,10,11,12,13^ Interestingly, the association between PP and poor cancer outcomes appears to be independent of patients’ current income level, suggesting additional causal mechanisms that may not be targeted by traditional welfare programs (eg, exposure to carcinogens, limited or delayed access to health care, and structural barriers to treatment adherence).^12^

The Biden–Harris Administration launched the Persistent Poverty Initiative through the National Cancer Institute in an effort to identify and alleviate the effects of PP on cancer outcomes.^10^ We sought to compare clinical outcomes among patients with colon cancer based on their degree of exposure to PP and then to explore 2 potential pathways that may mediate the association between PP and cancer-specific mortality. Such findings would improve our understanding of how exposure to PP affects patients with colon cancer in ways that can inform future policy interventions.

## Methods

### Patient Population

In this retrospective cohort study, we identified all patients with a new diagnosis of colon cancer between 2017 and 2020 from the California Cancer Registry, the largest statewide cancer registry in the US. We excluded patients with (1) more than 1 reported malignant tumor, (2) carcinoma in situ, (3) histologic findings other than adenocarcinoma, and (4) a diagnosis based only on the death certificate. We also excluded patients with a missing date of diagnosis, cancer stage, or zip code, as each of these variables was necessary to calculate our primary outcome. Our study conformed to the Strengthening the Reporting of Observational Studies in Epidemiology (STROBE) reporting guideline. The study was determined to be exempt from review by Stanford University’s Institutional Review Board as it involved existing, publicly available, and deidentified data. Informed consent was waived because the data were fully deidentified, preventing participant contact.

### Persistent Poverty

We used published data from the National Cancer Institute to identify US census tracts that met the definition of PP.^9,14^ Since census tract identifications were not available in our subset of California Cancer Registry data, we used a crosswalk from the US Department of Housing and Urban Development to link census tracts to 5-digit zip codes and then calculated the percentage of census tracts within each zip code that met criteria for PP (hereafter referred to as the PP ratio).^15^ For example, a zip code with 5 census tracts in which 4 met the criteria for PP would have a PP ratio of 0.80.

We categorized patients into 4 groups based on the PP ratio of their zip code at the time of their diagnosis: 0, 0.01 to 0.25, 0.26 to 0.50, and higher than 0.50. Since the categorization of zip codes by PP ratio has not been published previously, we conducted 2 sensitivity analyses. First, although census tracts contain roughly the same number of individuals, we calculated population-weighted PP ratios by dividing the number of individuals in each zip code living in PP by the total number of individuals.^16^ Second, we grouped zip codes by quartiles of PP ratios instead of our predefined categories. Since neither of these changes altered our results, we chose to report our original specification.

### Outcomes

Our primary outcome was disease-specific mortality. Patients were classified as alive, dead from colon cancer, or dead from another cause based on the Surveillance, Epidemiology, and End Results Program cause-specific death classification; patients with an unknown cause of death were treated as missing for the purpose of disease-specific mortality (n = 98 patients [0.5%]).^17^ Our secondary outcomes were overall mortality, cancer stage at diagnosis, and receipt of guideline-concordant care.

### Access and Quality

We explored differences in access to health care and quality of care as potential mediators of the association between living in PP and disease-specific mortality. We chose disease-specific mortality rather than overall mortality, as we believed that the former would be the most affected by potential differences in cancer-related treatment. We used local health care practitioner density as a measure of potential access to care. We calculated the number of primary care physicians, gastroenterologists, and general surgeons per 100 000 residents in each county based on the Area Health Resource Files from the Health Resources and Services Administration. For quality of care, we used guidelines from the National Comprehensive Cancer Network and timeliness of care measures from the National Quality Forum.^18,19,20^ We considered patients to be guideline concordant if they underwent definitive surgery (cancer stage I-II), underwent definitive surgery followed by systemic chemotherapy within 120 days (cancer stage III), or received systemic therapy (cancer stage IV).

### Statistical Analysis

In univariate analyses, categorical variables were compared using the Pearson χ^2^ test, and continuous variables were compared using analysis of variance. We used Fine-Gray competing risk proportional hazards regression models to calculate disease-specific mortality.^21^ Compared with traditional survival models, competing risk models allow for more complete use of survival data and prevent overestimation of the cumulative risk of death due to other causes.^22^ We used days from cancer diagnosis to either death or unavailability for follow-up as our time variable and treated death from another cause as a competing event. For overall survival, we used Cox proportional hazards models with the same time variable and censoring criteria. We also used multivariable logistic regression to assess the association between the PP ratio and the likelihood of having advanced disease at diagnosis and of receiving guideline discordant care. We excluded patients with cancer stage III disease who either died (n = 267 patients [1.3%]) or were unavailable for follow up (n = 112 patients [0.6%]) within 120 days of surgery from our assessment of guideline concordance, as they may not have had the opportunity to undergo adjuvant chemotherapy. We tested the association between living in PP and our outcome measures based on the significance of subdistribution hazard ratios (HRs) in our Fine-Gray models, HRs in our Cox model, and odds ratios in our logistic models.

All models controlled for patient-level demographic (age, race, ethnicity, sex, and rurality) and clinical (Charlson comorbidity index, cancer stage) variables as well as year of diagnosis. Charlson comorbidity index ranged from 0 to 14, with higher values indicating higher comorbidity. Race and ethnicity were assessed because of their close association with social determinants of health and their potential to contribute to clinical outcomes and were defined by the California Cancer Registry, which uses a combination of direct and indirect identification strategies.^23^ Race and ethnicity categories comprised Asian or Pacific Islander, Hispanic, Middle Eastern, non-Hispanic American Indian, non-Hispanic Black, non-Hispanic White, and other, which included patients with missing race or those who self-identified as a race not captured by the existing categories. Our model assessing the likelihood of initiating systemic therapy among patients with cancer stage IV disease also controlled for the pattern of metastatic disease (liver only, lung only, liver and lung, bone or brain, other). To prevent overfitting, we excluded variables with high degrees of multicollinearity (variance inflation factor >30), which included primary source of health insurance. As an additional sensitivity analysis, we stratified patients by their primary source of health insurance (Medicare, Medicaid, or private) to determine if there was a potential interaction between living in PP and insurance status. We used cluster-robust SEs at the hospital level in all regression analyses to account for unmeasured differences between treatment centers.

We used nested Fine-Gray models to assess access and quality of care as potential mediators of the association between PP and disease-specific mortality. We began with a base model controlling only for demographic and clinical characteristics (model 1) and then sequentially added measures of access (model 2) and quality of care (model 3). We interpreted changes to the significance of the association between PP and disease-specific mortality as evidence of potential mediation (ie, difference method).^24^ We used the McFadden pseudo-*R*^2^ to quantify the variability explained by our nested regression models. In Cox regression, pseudo-*R*^2^ provides a measure of how well the model fits the survival data. In survival data, since time censoring is included, pseudo-*R*^2^ tends to be lower than the traditional regression *R*^2^. Therefore, pseudo-*R*^2^ should be used to assess for model improvement compared with a baseline model. All statistical analyses were performed from February 2024 to February 2025 using Stata/SE, version 18.0 (StataCorp LLC). A 2-sided value of *P* < .05 was considered statistically significant.

## Results

**Figure 1.**
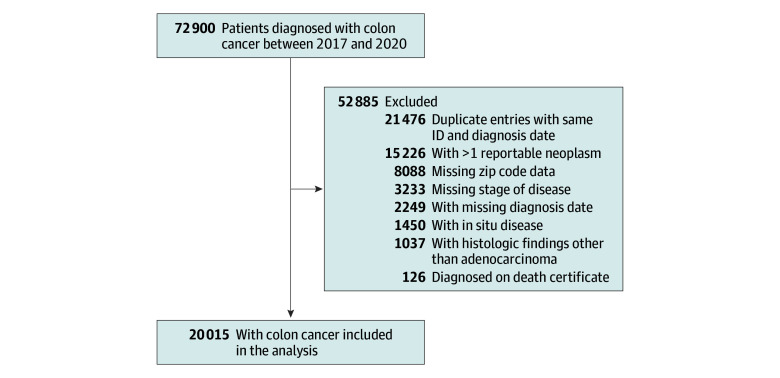
Flowchart of the Study Inclusion and Exclusion Criteria ID indicates identification number.

We identified 20 015 patients (48.7% female and 51.3% male) diagnosed with colon cancer in California between 2017 and 2020 (Figure 1). The mean (SD) age at diagnosis was 65.9 (14.0) years and 14.5% were Asian/Pacific Islander, 24.4% were Hispanic, 2.2% were Middle Eastern, 0.8% were non-Hispanic American Indian, 6.9% were non-Hispanic Black, 50.4% were non-Hispanic White, and 0.9% were other race and ethnicity. The majority (66.3%) lived in a zip code with no PP, and only 3.6% lived in a zip code with a PP ratio higher than 0.50. Patients living in areas with higher PP ratios were more likely to be younger (mean [SD] age at diagnosis, 64.3 [14.1] years for >50% PP vs 66.3 [14.1] years for no PP), to identify as Hispanic (45.5% for >50% PP vs 19.2% for no PP) or non-Hispanic Black (15.7% for >50% PP vs 4.9% for no PP), to use Medicaid as their primary source of insurance (25.1% for >50% PP vs 9.9% for no PP), and to have a higher Charlson comorbidity index (mean [SD] score, 1.3 [1.8] for >50% PP vs 1.2 [1.7] for no PP) (Table 1). Most patients (74.8%) had resectable disease at the time of diagnosis (cancer stages I-III). Compared with patients living in areas with no PP, patients living in zip codes with a PP ratio higher than 0.50 had a significantly higher likelihood of presenting with metastatic disease (odds ratio, 1.23 [95% CI, 1.02-1.47]) (eTable 1 in Supplement 1). The median (range) follow-up for patients in our cohort was 28 (0-72) months.

**Table 1.** Demographic and Clinical Characteristics of California Residents Diagnosed With Colon Cancer Based on the Density of Persistent Poverty in Their Zip Code

Characteristic	No. (%) of California residents with persistent poverty				*P* value
	None	≤25% CT	26%-50% CT	>50% CT	
No. of patients	13 260	4610	1429	716	NA
Age at diagnosis, mean (SD), y	66.3 (14.1)	65.5 (13.8)	65.0 (13.4)	64.3 (14.1)	<.001
Race and ethnicity					
Asian or Pacific Islander	2055 (15.5)	575 (12.5)	171 (12.0)	102 (14.3)	<.001
Hispanic	2546 (19.2)	1448 (31.4)	552 (38.7)	325 (45.5)	
Middle Eastern	302 (2.3)	117 (2.5)	26 (1.8)	2 (0.3)	
Non-Hispanic American Indian	89 (0.7)	38 (0.8)	13 (0.9)	12 (1.7)	
Non-Hispanic Black	655 (4.9)	450 (9.8)	158 (11.1)	112 (15.7)	
Non-Hispanic White	7478 (56.4)	1950 (42.3)	500 (35.0)	157 (22.0)	
Other or unknown^a^	129 (1.0)	32 (0.7)	8 (0.6)	5 (0.7)	
Sex					
Female	6497 (49.0)	2232 (48.4)	692 (48.5)	330 (46.2)	.46
Male	6757 (51.0)	2378 (51.6)	736 (51.5)	385 (53.8)	
Primary payment method					
Uninsured or self pay	86 (0.7)	43 (1.0)	12 (0.9)	9 (1.3)	<.001
Private plan, HMO, PPO	5172 (40.6)	1673 (37.4)	446 (32.7)	192 (27.5)	
Medicaid	1259 (9.9)	701 (15.7)	277 (20.3)	175 (25.1)	
Medicare	6045 (47.4)	2005 (44.9)	615 (45.1)	315 (45.1)	
Other or unknown	179 (1.4)	47 (1.1)	15 (1.1)	7 (1.0)	
Location					
Urban	11 312 (85.3)	4172 (90.5)	1008 (70.5)	578 (80.7)	<.001
Rural	1948 (14.7)	438 (9.5)	421 (29.5)	138 (19.3)	
Charlson comorbidity score, mean (SD)^b^	1.2 (1.7)	1.4 (1.8)	1.4 (1.7)	1.3 (1.8)	<.001
AJCC stage					
I	2746 (20.7)	904 (19.6)	269 (18.8)	119 (16.6)	.02
II	3489 (26.3)	1207 (26.2)	365 (25.5)	172 (24.0)	
III	3772 (28.4)	1311 (28.4)	409 (28.6)	214 (29.9)	
IV	3253 (24.5)	1188 (25.8)	386 (27.0)	211 (29.5)	
Year of diagnosis					
2017	3641 (27.5)	1293 (28.0)	377 (26.4)	197 (27.5)	.55
2018	3395 (25.6)	1169 (25.4)	363 (25.4)	185 (25.8)	
2019	3335 (25.2)	1186 (25.7)	380 (26.6)	199 (27.8)	
2020	2889 (21.8)	962 (20.9)	309 (21.6)	135 (18.9)	

Abbreviations: AJCC, American Joint Committee on Cancer; CT, census tract; HMO, health maintenance organization; NA, not applicable; PPO, preferred provider organization.

^a^ Other race and ethnicity included patients with missing race or those who self-identified as a race not captured by the existing categories.

^b^ Charlson comorbidity index ranged from 0 to 14, with higher values indicating higher comorbidity.

### Disease-Specific Mortality

**Figure 2.**
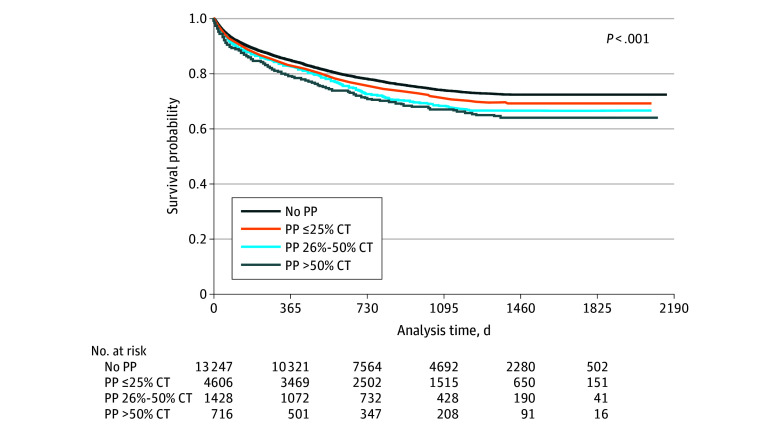
Disease-Specific Survival With Unadjusted *P* Value Based on the Level of Persistent Poverty (PP) Kaplan-Meier survival estimates. CT indicates census tract.

In unadjusted analyses, patients living in areas with higher PP ratios had a stepwise increase in both disease-specific mortality (19.5% for no PP, 21.5% for ≤25% PP, 23.9% for 26%-50% PP, and 24.8% for >50% PP) and overall mortality (Figure 2) (eTable 2 in Supplement 1). There were 5044 deaths in our patient cohort during the study period, with the vast majority (80.9%) being attributed to colon cancer.

Higher PP ratios were associated with higher risk of disease-specific mortality even after adjusting for patient-level characteristics (Table 2). Living in zip codes with PP ratios of 0.26 or higher was associated with a 19% (subdistribution HR for >50% PP, 1.19 [95% CI, 1.01-1.42]) to 20% (subdistribution HR for 26%-50% PP, 1.20 [95% CI, 1.07-1.36]) increase in the risk of colon cancer–related death compared with patients not living in PP. Increasing age (subdistribution HR, 1.03 [95% CI, 1.02-1.03]), comorbidity index (subdistribution HR, 1.12 [95% CI, 1.10-1.15]), and cancer stage (eg, for stage IV, subdistribution HR, 27.60 [95% CI, 23.30-32.68]) were also associated with higher risk of disease-specific mortality. After stratifying by insurance type, we found a persistent association between exposure to PP and higher risk of disease-specific mortality among patients enrolled in Medicare (HR, 1.12 [95% CI, 1.02-1.23]) or Medicaid (HR, 1.22 [95% CI, 1.02-1.46]), but not among patients with private insurance (HR, 1.02 [95% CI, 0.89-1.16]) (eTable 3 in in Supplement 1). Our results were similar when comparing overall as opposed to disease-specific mortality (eTable 4 in Supplement 1). Our results were also maintained when specifying PP ratio in quartiles rather than our predefined categories (eTables 5-6 in Supplement 1).

**Table 2.** Competing Risk Proportional Hazard Regression Models Estimating Disease-Specific Mortality

Disease-specific mortality	Subdistribution hazard ratio (95% CI)^a^	*P* value
Persistent poverty (reference: no persistent poverty)		
Persistent poverty ≤25% CT	1.07 (0.99-1.16)	.08
Persistent poverty 26%-50% CT	1.20 (1.07-1.36)	.003
Persistent poverty >50% CT	1.19 (1.01-1.42)	.04
Age at diagnosis, y	1.03 (1.02-1.03)	<.001
Race (reference: non-Hispanic White)		
Asian or Pacific Islander	0.93 (0.85-1.03)	.15
Hispanic	0.99 (0.91-1.08)	.83
Middle Eastern	0.68 (0.52-0.88)	.003
Non-Hispanic American Indian	0.81 (0.54-1.19)	.28
Non-Hispanic Black	1.09 (0.96-1.24)	.18
Other or unknown^b^	0.54 (0.32-0.93)	.03
Female sex (reference: male sex)	1.02 (0.96-1.09)	.52
Rural location (reference: urban)	1.05 (0.96-1.15)	.31
Charlson comorbidity index^c^	1.12 (1.10-1.15)	<.001
AJCC stage (reference: stage I)		
II	2.14 (1.77-2.59)	<.001
III	3.97 (3.32-4.74)	<.001
IV	27.60 (23.30-32.68)	<.001
Year of diagnosis (reference: 2017)		
2018	0.86 (0.79-0.93)	<.001
2019	0.68 (0.62-0.74)	<.001
2020	0.49 (0.44-0.56)	<.001

Abbreviations: AJCC, American Joint Committee on Cancer; CT, census tract.

^a^ Primary payment method was not included in the model due to multicollinearity (variance inflation factor >10).

^b^ Other race and ethnicity included patients with missing race or those who self-identified as a race not captured by the existing categories.

^c^ Charlson comorbidity index ranged from 0 to 14, with higher values indicating higher comorbidity.

### Access and Quality of Care

Areas with higher PP ratios had lower densities of primary care physicians, gastroenterologists, and general surgeons per 100 000 residents (eTable 7 in Supplement 1). Most patients across all stages received guideline-concordant care (82.7%). Living in PP was associated with a lower likelihood of receiving guideline-concordant care (eTable 8 in Supplement 1). There was no association between living in PP and the likelihood of undergoing definitive surgery among patients with stage I through III disease (eTable 9 in Supplement 1). However, higher PP ratios were associated with a stepwise decrease in the likelihood of initiating systemic therapy among patients with stage IV disease (odds ratios, 0.53-0.69) (eTable 10 in Supplement 1).

The results of our nested regression analysis are shown in Table 3. We found no significant difference in the association between PP and disease-specific mortality after controlling for health care practitioner density (model 2). However, we did find a difference in both the magnitude and the statistical significance of the association after controlling for receipt of guideline-concordant care, suggesting potential mediation (model 3) (<25% PP: model 1 subdistribution HR, 1.10 [1.01-1.20] vs model 3 subdistribution HR, 1.05 [0.96-1.15]; 26-50% PP: model 1 subdistribution HR, 1.24 [1.11-1.39] vs model 3 subdistribution HR, 1.15 [1.01-1.30]; >50% PP: model 1 subdistribution HR, 1.19 [0.98-1.44] vs model 3 subdistribution HR, 1.11 [0.91-1.35]). eTable 11 in Supplement 1 shows the results of the nested regression analysis after specifying PP ratios in quartiles.

**Table 3.** Nested Regression Analysis for Disease-Specific Mortality Based on the Preoperative and Postoperative Factors Analyzed

Disease-specific mortality^b^	Model 1^a^		Model 2^a^		Model 3^a^	
	Subdistribution hazard ratio (95% CI)	*P* value	Subdistribution hazard ratio (95% CI)	*P* value	Subdistribution hazard ratio (95% CI)	*P* value
Persistent poverty (reference: no persistent poverty)						
Persistent poverty ≤25% CT	1.10 (1.01-1.20)	.04	1.09 (1.00-1.19)	.04	1.05 (0.96-1.15)	.32
Persistent poverty 26%-50% CT	1.24 (1.11-1.39)	<.001	1.24 (1.10-1.39)	<.001	1.15 (1.01-1.30)	.03
Persistent poverty >50% CT	1.19 (0.98-1.44)	.08	1.18 (0.98-1.43)	.08	1.11 (0.91-1.35)	.29
Age at diagnosis	1.03 (1.02-1.03)	<.001	1.03 (1.02-1.03)	<.001	1.02 (1.01-1.02)	<.001
Race (reference: non-Hispanic White)						
Asian or Pacific Islander	0.94 (0.84-1.06)	.34	0.97 (0.86-1.09)	.57	0.96 (0.85-1.08)	.49
Hispanic	0.99 (0.91-1.08)	.79	0.99 (0.90-1.08)	.77	0.95 (0.86-1.05)	.30
Middle Eastern	0.65 (0.50-0.85)	.001	0.66 (0.51-0.86)	.002	0.68 (0.53-0.87)	.002
Non-Hispanic American Indian	0.71 (0.46-1.10)	.12	0.70 (0.45-1.09)	.11	0.72 (0.45-1.16)	.18
Non-Hispanic Black	1.11 (0.98-1.26)	.11	1.12 (0.98-1.27)	.09	1.08 (0.94-1.24)	.26
Other or unknown^c^	0.57 (0.33-0.98)	.04	0.57 (0.33-0.98)	.04	0.48 (0.27-0.86)	.01
Female sex (reference: male sex)	1.02 (0.96-1.10)	.48	1.02 (0.96-1.10)	.47	1.00 (0.93-1.07)	.99
Rural location (reference: urban)	1.05 (0.94-1.17)	.40	1.02 (0.91-1.14)	.78	1.04 (0.93-1.18)	.48
Charlson comorbidity index^d^	1.12 (1.10-1.15)	<.001	1.12 (1.10-1.15)	<.001	1.08 (1.06-1.11)	<.001
AJCC stage (reference: stage I)						
II	2.15 (1.76-2.62)	<.001	2.15 (1.77-2.62)	<.001	2.33 (1.92-2.83)	<.001
III	3.04 (2.51-3.68)	<.001	3.04 (2.51-3.69)	<.001	1.92 (1.58-2.34)	<.001
IV	28.05 (23.67-33.23)	<.001	28.07 (23.68-33.27)	<.001	20.51 (17.29-24.33)	<.001
Year of diagnosis (reference: 2017)						
2018	0.85 (0.78-0.92)	<.001	0.85 (0.78-0.92)	<.001	0.85 (0.78-0.93)	<.001
2019	0.69 (0.63-0.75)	<.001	0.68 (0.63-0.75)	<.001	0.70 (0.63-0.77)	<.001
2020	0.48 (0.42-0.55)	<.001	0.48 (0.42-0.55)	<.001	0.50 (0.44-0.56)	<.001
PCPs per 100 000 residents	NA	NA	1.00 (1.00-1.00)	.67	1.00 (1.00-1.00)	.11
Gastroenterologists per 100 000 residents	NA	NA	1.00 (0.96-1.04)	.99	1.00 (0.96-1.04)	.93
General surgeons per 100 000 residents	NA	NA	0.98 (0.97-1.00)	.08	0.98 (0.96-1.00)	.08
Guideline-discordant care	NA	NA	NA	NA	2.99 (2.72-3.30)	<.001
McFadden pseudo-*R*^2^, %	8.42	NA	8.43	NA	9.50	NA

Abbreviations: AJCC, American Joint Committee on Cancer; CT, census tract; NA, not applicable; PCPs, primary care physicians.

^a^ A base model controlled only for demographic and clinical characteristics (model 1) and then sequentially added measures of access (model 2) and quality of care (model 3).

^b^ Primary payment method was not included in the model due to multicollinearity (variance inflation factor >10).

^c^ Other race and ethnicity included patients with missing race or those who self-identified as a race not captured by the existing categories.

^d^ Charlson comorbidity index ranged from 0 to 14, with higher values indicating higher comorbidity.

## Discussion

In this retrospective cohort study, we found high hazards and consistent associations between exposure to PP and higher rates of disease-specific mortality among California residents diagnosed with colon cancer. We also found lower levels of health care practitioner density—a measure of access to care—and lower likelihoods of initiation of chemotherapy—a measure of quality of care—among patients living in PP. Based on our nested models, differences in quality of care appeared to primarily mediate the association between PP and higher rates of disease-specific mortality, suggesting that improving compliance with guideline-concordant care may reduce disparities in both care and clinical outcome. To our knowledge, ours is the first study to demonstrate a dose-response association for the association between PP and cancer-related mortality or to identify potential mechanisms.

Zip codes with higher shares of PP had both lower health care practitioner densities and higher rates of diagnosis at an advanced stage of disease, suggesting limited access to care. Multiple studies have demonstrated similar findings in rural areas—which tend to have higher rates of PP—including lower health care practitioner densities, higher rates of advanced stage at diagnosis, and higher case-fatality rates among patients with gastrointestinal cancers.^25,26^ Although limited availability of screening and early detection services has been hypothesized as a potential mechanism, several studies, including ours, demonstrated persistent disparities in clinical outcome even after controlling for structural measures of access.^26,27^ Several explanations may explain this apparent disconnect. First, health care practitioner density is only partial correlated with the availability of services at the individual level, which is also affected by geography, insurance status, and out-of-pocket costs.^4,28,29^ Second, potential access may not translate into realized access if patients either do not understand their health care needs or have alternative health beliefs. Studies comparing patients with cancer in rural and urban counties have documented differences in health care–seeking behaviors and higher levels of nonadherence to screening recommendations among rural communities.^30,31^ Although our results cannot determine why there is no apparent association between health care practitioner density, cancer stage at diagnosis, and cancer-specific mortality, they do suggest that simply increasing the number of available health care practitioner in areas of PP may not improve access or clinical outcomes.

Differences in guideline compliance, on the other hand, did appear to mediate the association between living in PP and cancer-specific mortality. Although we found no association between living in a zip code with a larger share of PP and the likelihood of undergoing definitive surgery, which is in line with previous studies that explored PP at the county level,^4^ we did find an association between higher PP ratios and lower likelihoods of initiating systemic treatment. Prior studies have demonstrated an association between living in a low-income zip code and lower rates of systemic treatment for colorectal cancer^32,33,34^; our study extends these findings to patients living in zip codes with a larger share of PP. Perhaps more interestingly, the association between living in PP and disease-specific mortality was no longer apparent after controlling for guideline compliance, suggesting that differences in oncologic outcomes may be mediated by differences in the quality of oncologic care. Again, several potential explanations exist. First, patients living in areas of PP may be less likely to be offered guideline-concordant care at both an individual level and a systems level. Patients living in PP areas tend to receive care at lower-volume centers, which are less likely to provide guideline-concordant therapies.^35,36^ Implicit bias may also play a role, especially considering prior data demonstrating differences in the receipt of adjuvant therapy between Black and White patients despite no difference in the likelihood of referral to either medical or radiation oncologists.^37^ Second, structural barriers, such as transportation insecurity or caregiving responsibilities, may prevent patients living in areas of PP from receiving guideline-concordant care.^38^ Finally, patients living in areas of PP may be less likely to understand or accept guideline-concordant therapies. Living in PP has been associated with a higher level of discrimination and distrust in the medical system, which may lead even well-informed patients to choose not to undergo treatment.^29,39,40^ Whatever the source, the association between PP and the receipt of cancer care appears to be complex, pervasive, and unlikely to be reversed through distributive welfare programs alone.^41,42^

Although our results demonstrate a dose-response association such that higher PP ratios were generally associated with higher rates of disease-specific mortality, this was not consistent, linear, or homogeneous throughout the cohort. Zip codes with PP ratios of 0.26 through 0.50 and higher than 0.50 both had significantly higher disease-specific mortality compared with zip codes without PP; however, their subdistribution HRs could not be distinguished from one another in either our primary or our mediation analyses. This result may be due to the small proportion of patients living in zip codes with a PP ratio higher than 0.50 (only 3.6% of the cohort), although it may also suggest a threshold relationship such that once an area reaches a certain level of PP, it is more difficult for residents to overcome or escape its effects. Multiple prior studies have also examined the potential interaction between individual-level sociodemographic factors and neighborhood disadvantage, generally finding independent but additive associations with cancer outcomes.^43,44,45,46^ Our study extends these findings by demonstrating higher disease-specific mortality among patients with public insurance (ie, Medicare or Medicaid) who are living in PP, but not among patients with private insurance who live in similar zip codes. Although the mechanisms remain unknown, private insurance—or the sociodemographic differences associated with having private as opposed to public insurance—may facilitate better access to better cancer care networks, thereby allowing patients to escape at least some of the deleterious effects of living in PP.

Our study has important implications regarding efforts to improve cancer health equity. The association between living in PP and higher rates of disease-specific mortality appeared to be concentrated among patients enrolled in public insurance, suggesting that the effects of PP may be moderated by other SDoHs. Studies continue to demonstrate the multiplicative effects of social barriers on cancer care and cancer outcomes, raising the concern that efforts to address SDoHs in isolation may be both inefficient and inadequate.^5,47,48^ Although targeted interventions do appear to improve health and reduce health care costs, the benefits of these programs are often diffuse and may accrue to institutions other than the one making the initial investment (ie, the wrong pocket problem).^42^ The Persistent Poverty Initiative represents a unique opportunity to address both of these limitations. First, the initiative can help establish PP as an independent SDoH and fund research to uncover mechanisms connecting SDoHs and the receipt of guideline-concordant cancer care. Second, as we build the necessary evidence, the initiative can support interventions designed to help patients with cancer living in PP overcome known barriers to care (eg, transportation insecurity), including by sponsoring high-risk high-reward projects that may otherwise go unfunded.

### Limitations

Our results should be interpreted with respect to several limitations. First, since census tract identifications were not available in our data, we generated an alternative measure of PP at the zip code level that has not been previously validated. Using PP ratios enabled us to compare geographic areas based on their share of PP and to identify a dose-response association, which has not, to our knowledge, been reported in previous literature. To the extent that PP ratios at the zip code level are less sensitive than measuring PP at the census tract level, we would expect our results to be conservative. Our measure also represents the proportion of census tracts per zip code that are in PP rather than the proportion of individuals. Given that census tracts are designed to have roughly similar populations—and that we found similar results when using population-weighted PP ratios—we do not believe that variation in the size of census tracts significantly affected our results. Still, the density of PP within a zip code may have an independent association with cancer care and mortality that is worth further study. Second, our results can only be interpreted at the zip code level rather than at the level of the individual patient (ie, the risk of ecological fallacy). Third, our data do not allow for an assessment of why patients living in PP had lower rates of guideline-concordant care, particularly whether patients chose not to undergo certain services or services were not offered to them. We focused on the availability of primary care physicians, gastroenterologists, and general surgeons as potential proxies for the availability of cancer screening or the capacity to evaluate colon cancer–related symptoms, especially considering that other analyses have found these services to be lacking in areas of PP.^27^ The availability of other types of clinicians (eg, medical oncologists) may have also affected access to cancer-specific care in ways that are worth further analysis. Fourth, despite the best efforts of the cancer registry, some patients may have been unavailable for follow-up, which may affect our assessment of the quality of cancer care. Since our median follow-up was 28 months and most colon cancer-directed therapy occurs within 12 months of diagnosis, we do not believe this limitation to have significantly affected our results. Fifth, tumor grade may have affected treatment or survival but could not be accounted for due to high rates of missing data. In addition, our data represent patients diagnosed with colon cancer in California and may not generalize to other tumor types (including rectal cancer) and other geographic regions.

## Conclusions

Findings from this retrospective cohort study indicated that living in an area of PP is independently associated with disease-specific mortality among patients diagnosed with colon cancer. Although areas of PP have a lower density of health care practitioners, the association between PP and mortality appears to be mediated through lower receipt of guideline-concordant cancer care. Our ability to treat and even cure cancer continues to accelerate at an exponential pace; the Persistent Poverty Initiative presents a unique opportunity to improve our understanding of PP as an independent SDoH and to support efforts to extend the benefits of treatment to all US residents with cancer.

## Notes

Supplement 1. **eTable 1.** Logistic regression analysis for diagnosis at an advanced stage (stage IV vs. stages I-III) **eTable 2.** Outcomes of patients based on the density of persistent poverty in the zip code of residence **eTable 3.** Cox proportional hazard regression analysis for overall mortality and Fine-Gray competing risk proportional hazard regression analysis for disease specific mortality based on primary insurance in patients exposed to persistent poverty (grouped as a binary variable: Yes / No) **eTable 4.** Cox proportional hazard regression analysis for overall mortality **eTable 5.** Demographic and clinical characteristics of Californians diagnosed with colon cancer with persistent poverty grouped in quartiles **eTable 6.** Fine-Gray competing risk proportional hazard regression models predicting disease specific mortality (secondary analysis) **eTable 7.** Number of primary care physicians, gastroenterologists, and general surgeons per county **eTable 8.** Logistic regression analysis for likelihood of receiving guideline discordant care **eTable 9.** Logistic regression analysis for likelihood of undergoing definitive surgery in patients with stage I-III disease **eTable 10.** Logistic regression analysis for likelihood of initiation of systemic treatment in patients with stage IV disease **eTable 11.** Nested regression analysis for disease specific mortality based on the pre-operative and post-operative factors analyzed for the secondary analysis

Supplement 2.
Data Sharing Statement
