# Supplementary material for: Outcomes Among Patients With Colon Cancer Living in Neighborhoods With Persistent Poverty
Source: JAMA Netw Open. 2026 Jan 9;9(1):e2551212. doi: 10.1001/jamanetworkopen.2025.51212 (PMC12789951; doi:10.1001/jamanetworkopen.2025.51212)
Supplement: Supplement 1. — eTable 1. Logistic regression analysis for diagnosis at an advanced stage (stage IV vs. stages I-III) eTable 2. Outcomes of patients based on the density of persistent poverty in the zip code of residence eTable 3. Cox proportional hazard regression analysis for overall mortality and Fine-Gray competing risk proportional hazard regression analysis for disease specific mortality based on primary insurance in patients exposed to persistent poverty (grouped as a binary variable: Yes / No) eTable 4. Cox proportional hazard regression analysis for overall mortality eTable 5. Demographic and clinical characteristics of Californians diagnosed with colon cancer with persistent poverty grouped in quartiles eTable 6. Fine-Gray competing risk proportional hazard regression models predicting disease specific mortality (secondary analysis) eTable 7. Number of primary care physicians, gastroenterologists, and general surgeons per county eTable 8. Logistic regression analysis for likelihood of receiving guideline discordant care eTable 9. Logistic regression analysis for likelihood of undergoing definitive surgery in patients with stage I-III disease eTable 10. Logistic regression analysis for likelihood of initiation of systemic treatment in patients with stage IV disease eTable 11. Nested regression analysis for disease specific mortality based on the pre-operative and post-operative factors analyzed for the secondary analysis [file jamanetwopen-e2551212-s001.pdf]

Supplementary Online Content

Naar L, Pohl AL, Morris AM, Dawes AJ. Outcomes among patients with colon cancer living in persistent poverty neighborhoods. *JAMA Netw Open*. 2026;9(1):e2551212. doi:10.1001/jamanetworkopen.2025.51212

eTable 1. Logistic regression analysis for diagnosis at an advanced stage (stage IV vs. stages I-III)

eTable 2. Outcomes of patients based on the density of persistent poverty in the zip code of residence

eTable 3. Cox proportional hazard regression analysis for overall mortality and Fine-Gray competing risk proportional hazard regression analysis for disease specific mortality based on primary insurance in patients exposed to persistent poverty (grouped as a binary variable: Yes / No)

eTable 4. Cox proportional hazard regression analysis for overall mortality

eTable 5. Demographic and clinical characteristics of Californians diagnosed with colon cancer with persistent poverty grouped in quartiles

eTable 6. Fine-Gray competing risk proportional hazard regression models predicting disease specific mortality (secondary analysis)

eTable 7. Number of primary care physicians, gastroenterologists, and general surgeons per county

eTable 8. Logistic regression analysis for likelihood of receiving guideline discordant care

eTable 9. Logistic regression analysis for likelihood of undergoing definitive surgery in patients with stage I-III disease

eTable 10. Logistic regression analysis for likelihood of initiation of systemic treatment in patients with stage IV disease

eTable 11. Nested regression analysis for disease specific mortality based on the pre-operative and post-operative factors analyzed for the secondary analysis

This supplementary material has been provided by the authors to give readers additional information about their work.

© 2026 Naar L et al. *JAMA Network Open*.

eTable 1. Logistic regression analysis for diagnosis at an advanced stage (stage IV vs. stages I-III)

**Stage IV vs Stages I-III**	**Odds ratio**	**p-value**	**95% CI**
**Persistent poverty (Reference: no persistent poverty)**			
Persistent poverty ≤25% CT	1.03	0.49	0.94-1.14
Persistent poverty 26%-50% CT	1.11	0.18	0.96-1.28
Persistent poverty >50% CT	1.23	0.03	1.02-1.47
**Age at diagnosis**	0.99	<0.001	0.98-0.99
**Race (Reference: Non-Hispanic White)**			
Asian/Pacific Islander	1.01	0.83	0.91-1.13
Hispanic	1.04	0.36	0.95-1.15
Middle Eastern	0.98	0.88	0.78-1.24
Non-Hispanic American Indian	1.04	0.84	0.70-1.57
Non-Hispanic Black	1.35	<0.001	1.20-1.51
Other/Unknown	0.68	0.05	0.47-0.99
**Sex (Reference: Male)**	0.91	0.01	0.85-0.98
**Charlson comorbidity score**	1.02	0.16	0.99-1.04
**Year of diagnosis (Reference: 2017)**			
2018	1.03	0.52	0.94-1.13
2019	0.98	0.73	0.90-1.08
2020	1.05	0.34	0.95-1.18

CI: confidence interval, CT: census tract

"Primary payment method" was not included in the model due to multicollinearity (vif>10).

© 2026 Naar L et al. *JAMA Network Open*.

eTable 2. Outcomes of patients based on the density of persistent poverty in the zip code of residence

	No persistent poverty	Persistent poverty ≤25% CT	Persistent poverty 26-50% CT	Persistent poverty >50% CT	p-value
**Number of patients**	13,260	4,610	1,429	716	
**Disease specific mortality**	2,580 (19.5%)	988 (21.5%)	338 (23.9%)	176 (24.8%)	<0.001
**Overall mortality**	3,192 (24.1%)	1,218 (26.4%)	414 (29.0%)	220 (30.7%)	<0.001

© 2026 Naar L et al. *JAMA Network Open*.

eTable 3. Cox proportional hazard regression analysis for overall mortality and Fine-Gray competing risk proportional hazard regression analysis for disease specific mortality based on primary insurance in patients exposed to persistent poverty (grouped as a binary variable: Yes / No)

	Hazard Ratio*	p-value
Private Insurance		
Disease specific mortality	1.02	0.81
Overall mortality	1.04	0.47
Medicare insurance		
Disease specific mortality	1.12	0.02
Overall mortality	1.10	0.02
Medicaid Insurance		
Disease specific mortality	1.22	0.03
Overall mortality	1.24	0.005

*The results reported in this table are adjusted hazard ratios for the following covariates: age, race, sex, urban/rural location, Charlson comorbidity index, stage of cancer at diagnosis, year of diagnosis

© 2026 Naar L et al. *JAMA Network Open*.

eTable 4. Cox proportional hazard regression analysis for overall mortality

**Overall mortality**	**Hazard Ratio**	**p-value**	**95% CI**
**Persistent poverty (Reference: no persistent poverty)**			
Persistent poverty ≤25% CT	1.07	0.08	0.99-1.15
Persistent poverty 26%-50% CT	1.20	<0.001	1.09-1.32
Persistent poverty >50% CT	1.24	0.02	1.04-1.48
**Age at diagnosis**	1.03	<0.001	1.03-1.04
**Race (Reference: Non-Hispanic White)**			
Asian/Pacific Islander	0.92	0.07	0.84-1.01
Hispanic	1.02	0.60	0.95-1.10
Middle Eastern	0.73	0.004	0.59-0.90
Non-Hispanic American Indian	0.77	0.21	0.51-1.16
Non-Hispanic Black	1.08	0.21	0.96-1.22
Other/Unknown	0.67	0.07	0.43-1.04
**Sex (Reference: Male)**	0.99	0.74	0.93-1.05
**Rural location (Reference: Urban)**	1.06	0.27	0.96-1.17
**Charlson comorbidity score**	1.20	<0.001	1.18-1.22
**AJCC Stage (Reference: Stage I)**			
Stage II	1.55	<0.001	1.36-1.76
Stage III	2.38	<0.001	2.10-2.71
Stage IV	14.38	<0.001	12.88-16.06
**Year of diagnosis (Reference: 2017)**			
2018	0.84	<0.001	0.78-0.91
2019	0.66	<0.001	0.60-0.71
2020	0.52	<0.001	0.46-0.58

CI: confidence interval, CT: census tract, AJCC: American Joint Committee on Cancer

"Primary payment method" was not included in the model due to multicollinearity (vif>10).

© 2026 Naar L et al. *JAMA Network Open*.

eTable 5. Demographic and clinical characteristics of Californians diagnosed with colon cancer with persistent poverty grouped in quartiles

	No persistent poverty	Persistent poverty Q1	Persistent poverty Q2	Persistent poverty Q3	Persistent poverty Q4	p-value
**Number of patients**	13,260	1,502	1,880	1,593	1,780	
**Age at diagnosis, mean (SD)**	66.3 (14.1)	65.8 (13.3)	65.6 (14.4)	65.2 (13.5)	64.5 (13.7)	<0.001
**Race**						<0.001
Asian/Pacific Islander	2,055 (15.5%)	213 (14.2%)	195 (10.4%)	217 (13.6%)	223 (12.5%)	
Hispanic	2,546 (19.2%)	430 (28.6%)	609 (32.4%)	526 (33.0%)	760 (42.7%)	
Middle Eastern	302 (2.3%)	21 (1.4%)	67 (3.6%)	43 (2.7%)	14 (0.8%)	
Non-Hispanic American Indian	89 (0.7%)	15 (1.0%)	10 (0.5%)	15 (0.9%)	23 (1.3%)	
Non-Hispanic Black	655 (4.9%)	102 (6.8%)	213 (11.3%)	169 (10.6%)	236 (13.3%)	
Non-Hispanic White	7,478 (56.4%)	704 (46.9%)	775 (41.2%)	619 (38.9%)	509 (28.6%)	
Other/Unknown	129 (1.0%)	17 (1.1%)	11 (0.6%)	4 (0.3%)	13 (0.7%)	
**Sex**						0.40
Male	6,757 (51.0%)	762 (50.7%)	964 (51.3%)	823 (51.7%)	950 (53.4%)	
Female	6,497 (49.0%)	740 (49.3%)	916 (48.7%)	770 (48.3%)	828 (46.6%)	
**Primary payment method**						
Uninsured / self pay	86 (0.7%)	11 (0.8%)	19 (1.0%)	15 (1.0%)	19 (1.1%)	
Private plan, HMO, PPO	5,172 (40.6%)	565 (39.0%)	664 (36.4%)	565 (36.5%)	517 (30.2%)	
Medicaid	1,259 (9.9%)	202 (14.0%)	298 (16.3%)	258 (16.7%)	395 (23.1%)	
Medicare	6,045 (47.4%)	658 (45.4%)	823 (45.1%)	693 (44.8%)	761 (44.4%)	
Other/Unknown	179 (1.4%)	12 (0.8%)	20 (1.1%)	16 (1.0%)	21 (1.2%)	<0.001
**Urban / Rural location**						

© 2026 Naar L et al. *JAMA Network Open*.

Urban	11,312 (85.3%)	1,403 (93.4%)	1,756 (93.4%)	1,248 (78.3%)	1,351 (75.9%)	
Rural	1,948 (14.7%)	99 (6.6%)	124 (6.6%)	345 (21.7%)	429 (24.1%)	
**Charlson comorbidity score, mean (SD)**	1.2 (1.7)	1.3 (1.7)	1.4 (1.8)	1.4 (1.9)	1.3 (1.8)	<0.001
**AJCC Stage**						0.01
Stage I	2,746 (20.7%)	302 (20.1%)	377 (20.1%)	295 (18.5%)	318 (17.9%)	
Stage II	3,489 (26.3%)	393 (26.2%)	498 (26.5%)	424 (26.6%)	429 (24.1%)	
Stage III	3,772 (28.4%)	428 (28.5%)	537 (28.6%)	440 (27.6%)	529 (29.7%)	
Stage IV	3,253 (24.5%)	379 (25.2%)	468 (24.9%)	434 (27.2%)	504 (28.3%)	
**Year of diagnosis**						0.78
2017	3,641 (27.5%)	418 (27.8%)	513 (27.3%)	454 (28.5%)	482 (27.1%)	
2018	3,395 (25.6%)	386 (25.7%)	499 (26.5%)	379 (23.8%)	453 (25.4%)	
2019	3,335 (25.2%)	389 (25.9%)	488 (26.0%)	416 (26.1%)	472 (26.5%)	
2020	2,889 (21.8%)	309 (20.6%)	380 (20.2%)	344 (21.6%)	373 (21.0%)	

SD: standard deviation, AJCC: American Joint Committee on Cancer

© 2026 Naar L et al. *JAMA Network Open*.

eTable 6. Fine-Gray competing risk proportional hazard regression models predicting disease specific mortality (secondary analysis)

Disease specific mortality	Subdistribution Hazard Ratio	p-value	95% CI
**Persistent poverty (Reference: no persistent poverty)**			
Persistent poverty Q1	1.11	0.09	0.98 – 1.25
Persistent poverty Q2	1.05	0.39	0.94 – 1.18
Persistent poverty Q3	1.13	0.04	1.01 – 1.27
Persistent poverty Q4	1.16	0.009	1.04 – 1.30
**Age at diagnosis**	1.03	<0.001	1.02 – 1.03
**Race (Reference: Non-Hispanic White)**			
Asian/Pacific Islander	0.93	0.16	0.85 – 1.03
Hispanic	0.99	0.89	0.92 – 1.08
Middle Eastern	0.68	0.004	0.53 – 0.88
Non-Hispanic American Indian	0.80	0.27	0.54 – 1.19
Non-Hispanic Black	1.10	0.16	0.97 – 1.24
Other/Unknown	0.54	0.03	0.32 – 0.93
**Sex (Reference: Male)**	1.02	0.53	0.96 – 1.09
**Rural location (Reference: Urban)**	1.05	0.28	0.96 – 1.15
**Charlson comorbidity score**	1.12	<0.001	1.10 – 1.15
**AJCC Stage (Reference: Stage I)**			
Stage II	2.14	<0.001	1.77 – 2.59
Stage III	3.97	<0.001	3.32 – 4.75
Stage IV	27.58	<0.001	23.29 – 32.67
**Year of diagnosis (Reference: 2017)**			
2018	0.86	<0.001	0.80 – 0.93
2019	0.68	<0.001	0.62 – 0.74
2020	0.49	<0.001	0.44 – 0.56

CI: confidence interval, CT: census tract, AJCC: American Joint Committee on Cancer

"Primary payment method" was not included in the model due to multicollinearity (vif>10).

© 2026 Naar L et al. *JAMA Network Open*.

eTable 7. Number of primary care physicians, gastroenterologists, and general surgeons per county

	No persistent poverty	Persistent poverty ≤25% CT	Persistent poverty 26-50% CT	Persistent poverty >50% CT	p-value
**Number of patients**	13,260	4,610	1,429	716	
**PCPs per 100K, mean (SD)**	93.8 (29.0)	87.8 (30.7)	77.5 (30.1)	78.2 (21.7)	<0.001
**GI per 100K, mean (SD)**	4.4 (1.9)	4.2 (1.8)	3.4 (1.9)	3.5 (1.7)	<0.001
**General Surgeons per 100K, mean (SD)**	11.2 (4.0)	11.0 (4.5)	9.7 (4.1)	10.2 (2.9)	<0.001

CT: census tract, PCP: Primary care physicians, SD: standard deviation, GI: Gastroenterologists

© 2026 Naar L et al. *JAMA Network Open*.

eTable 8. Logistic regression analysis for likelihood of receiving guideline discordant care

**Guideline discordant care**	**Odds ratio**	**p-value**	**95% CI**
**Persistent poverty (Reference: no persistent poverty)**			
Persistent poverty ≤25% CT	1.27	<0.001	1.12 – 1.44
Persistent poverty 26%-50% CT	1.45	<0.001	1.22 – 1.74
Persistent poverty >50% CT	1.78	<0.001	1.40 – 2.26
**Age at diagnosis**	1.05	<0.001	1.05 – 1.06
**Race (Reference: Non-Hispanic White)**			
Asian/Pacific Islander	0.94	0.39	0.81 – 1.09
Hispanic	1.09	0.20	0.96 – 1.25
Middle Eastern	1.05	0.85	0.66 – 1.65
Non-Hispanic American Indian	1.13	0.66	0.66 – 1.91
Non-Hispanic Black	1.13	0.22	0.93 – 1.37
Other/Unknown	1.61	0.20	0.77 – 3.33
**Sex (Reference: Male)**	1.08	0.12	0.98 – 1.19
**Charlson comorbidity score**	1.19	<0.001	1.16 – 1.22
**AJCC Stage (Reference: Stage I)**			
Stage II	0.28	<0.001	0.21 – 0.38
Stage III	11.17	<0.001	9.00 – 13.86
Stage IV	11.91	<0.001	9.83 – 14.44

CI: confidence interval, CT: census tract, AJCC: American Joint Committee on Cancer

The following variables were not included in the model as they did not meet the set p-value threshold (<0.20) on univariate analysis: Sex.

"Primary payment method" was not included in the model due to multicollinearity (vif>10).

© 2026 Naar L et al. *JAMA Network Open*.

eTable 9. Logistic regression analysis for likelihood of undergoing definitive surgery in patients with stage I-III disease

Definitive surgery	Odds ratio	p-value	95% CI
**Persistent poverty (Reference: no persistent poverty)**			
Persistent poverty ≤25% CT	0.85	0.23	0.65-1.11
Persistent poverty 26%-50% CT	0.68	0.15	0.40-1.15
Persistent poverty >50% CT	0.63	0.14	0.35-1.16
**Age at diagnosis**	0.96	<0.001	0.94-0.97
**Race (Reference: Non-Hispanic White)**			
Asian/Pacific Islander	1.17	0.48	0.76-1.81
Hispanic	0.73	0.03	0.55-0.97
Middle Eastern	1.28	0.63	0.46-3.52
Non-Hispanic American Indian	0.59	0.36	0.19-1.84
Non-Hispanic Black	0.62	0.03	0.40-0.95
Other/Unknown	0.41	0.07	0.16-1.09
**Rural location (Reference: Urban)**	0.77	0.10	0.56-1.05
**Charlson comorbidity score**	0.87	<0.001	0.82-0.93
**AJCC Stage (Reference: Stage I)**			
Stage II	3.38	<0.001	2.51-4.55
Stage III	10.10	<0.001	6.64-15.35
**Year of diagnosis (Reference: 2017)**			
2018	0.90	0.57	0.64-1.28
2019	1.01	0.93	0.72-1.43
2020	0.72	0.05	0.51-1.00

CI: confidence interval, CT: census tract, AJCC: American Joint Committee on Cancer

The following variables were not included in the model as they did not meet the set p-value threshold (<0.20) on univariate analysis: Sex.

"Primary payment method" was not included in the model due to multicollinearity (vif>10).

© 2026 Naar L et al. *JAMA Network Open*.

eTable 10. Logistic regression analysis for likelihood of initiation of systemic treatment in patients with stage IV disease

Initiation of systemic treatment	Odds ratio	p-value	95% CI
**Persistent poverty (Reference: no persistent poverty)**			
Persistent poverty ≤25% CT	0.69	<0.001	0.58-0.82
Persistent poverty 26%-50% CT	0.56	<0.001	0.43-0.73
Persistent poverty >50% CT	0.53	0.002	0.36-0.79
**Age at diagnosis**	0.95	<0.001	0.94-0.95
**Race (Reference: Non-Hispanic White)**			
Asian/Pacific Islander	1.19	0.12	0.96-1.47
Hispanic	1.00	0.99	0.85-1.18
Middle Eastern	1.25	0.46	0.69-2.27
Non-Hispanic American Indian	1.27	0.52	0.62-2.59
Non-Hispanic Black	0.91	0.51	0.69-1.20
Other/Unknown	0.84	0.73	0.32-2.24
**Charlson comorbidity score**	0.81	<0.001	0.78-0.85
**Metastatic burden (Reference: Liver only)**			
Lung only	0.83	0.25	0.60-1.14
Liver and lung only	0.77	0.03	0.61-0.97
Bone or Brain only	0.17	<0.001	0.08-0.36
Other (including single or multiple foci)	0.69	<0.001	0.60-0.81

CI: confidence interval, CT: census tract

The following variables were not included in the model as they did not meet the set p-value threshold (<0.20) on univariate analysis: Sex, Year of diagnosis.

"Primary payment method" was not included in the model due to multicollinearity (vif>10).

© 2026 Naar L et al. *JAMA Network Open*.

eTable 11. Nested regression analysis for disease specific mortality based on the pre-operative and post-operative factors analyzed for the secondary analysis

Disease specific mortality	Subdistribution Hazard Ratio	p-value	95% CI	Subdistribution Hazard Ratio	p-value	95% CI	Subdistribution Hazard Ratio	p-value	95% CI
	Model 1			Model 2			Model 3		
**Persistent poverty (Reference: no persistent poverty)**									
Persistent poverty Q1	1.13	0.08	0.98 – 1.30	1.14	0.07	0.99 – 1.31	1.09	0.28	0.93 – 1.26
Persistent poverty Q2	1.06	0.35	0.94 – 1.21	1.05	0.48	0.92 – 1.19	1.00	0.99	0.88 – 1.14
Persistent poverty Q3	1.18	0.004	1.06 – 1.32	1.19	0.002	1.06 – 1.33	1.14	0.03	1.01 – 1.28
Persistent poverty Q4	1.17	0.004	1.05 – 1.31	1.17	0.006	1.05 – 1.30	1.09	0.17	0.96 – 1.23
**Age at diagnosis**	1.03	<0.001	1.02 – 1.03	1.03	<0.001	1.02 – 1.03	1.02	<0.001	1.01 -1.02
**Race (Reference: Non-Hispanic White)**									
Asian/Pacific Islander	0.94	0.33	0.84 – 1.06	0.97	0.58	0.86 – 1.09	0.96	0.49	0.85 – 1.08
Hispanic	0.99	0.85	0.91 – 1.08	0.99	0.84	0.91 – 1.08	0.95	0.34	0.87 – 1.05
Middle Eastern	0.66	0.002	0.50 – 0.85	0.67	0.002	0.51 – 0.87	0.69	0.003	0.54 – 0.88
Non-Hispanic American Indian	0.70	0.12	0.45 – 1.09	0.69	0.10	0.44 – 1.08	0.72	0.17	0.44 – 1.15
Non-Hispanic Black	1.12	0.10	0.98 – 1.27	1.13	0.07	0.99 – 1.28	1.09	0.22	0.95 – 1.24
Other/Unknown	0.57	0.04	0.33 – 0.98	0.56	0.04	0.33 – 0.97	0.48	0.01	0.27 – 0.86
**Sex (Reference: Male)**	1.02	0.50	0.96 – 1.09	1.02	0.48	0.96 – 1.09	1.00	0.99	0.93 – 1.07
**Rural location (Reference: Urban)**	1.05	0.37	0.94 – 1.18	1.02	0.80	0.90 – 1.14	1.04	0.50	0.92 – 1.17
**Charlson comorbidity score**	1.12	<0.001	1.10 – 1.15	1.12	<0.001	1.10 – 1.15	1.08	<0.001	1.06 – 1.11
**AJCC Stage (Reference: Stage I)**									
Stage II	2.15	<0.001	1.76 – 2.62	2.15	<0.001	1.77 – 2.62	2.33	<0.001	1.92 – 2.83
Stage III	3.04	<0.001	2.51 – 3.69	3.05	<0.001	2.51 – 3.69	1.92	<0.001	1.58 – 2.34
Stage IV	28.03	<0.001	23.65 – 33.22	28.06	<0.001	23.67 – 33.25	20.50	<0.001	17.28 – 24.31
**Year of diagnosis (Reference: 2017)**									
2018	0.85	<0.001	0.78 – 0.92	0.85	<0.001	0.78 – 0.92	0.86	<0.001	0.78 – 0.93

© 2026 Naar L et al. *JAMA Network Open*.

2019	0.69	<0.001	0.63 – 0.75	0.69	<0.001	0.63 – 0.75	0.70	<0.001	0.63 – 0.77
2020	0.48	<0.001	0.42 – 0.55	0.48	<0.001	0.43 – 0.55	0.50	<0.001	0.44 – 0.57
**PCPs per 100K**	-	-	-	1.00	0.63	1.00 – 1.00	1.00	0.09	0.99 – 1.01
**GI per 100K**	-	-	-	1.00	0.92	0.96 – 1.04	1.00	0.86	0.96 – 1.04
**General Surgeons per 100K**	-	-	-	0.98	0.07	0.97 – 1.00	0.98	0.07	0.96 – 1.00
**Guideline discordant care**	-	-	-	-	-	-	3.00	<0.001	2.72 – 3.30

© 2026 Naar L et al. *JAMA Network Open*.
