# Supplementary material for: Outcomes Among Patients With Colon Cancer Living in Neighborhoods With Persistent Poverty
Source: JAMA Netw Open. 2026 Jan 9;9(1):e2551212. doi: 10.1001/jamanetworkopen.2025.51212 (PMC12789951; doi:10.1001/jamanetworkopen.2025.51212)
Supplement: Supplement 2. — Data Sharing Statement [file jamanetwopen-e2551212-s002.pdf]

Data Sharing Statement

Naar. Outcomes Among Patients With Colon Cancer Living in Persistent Poverty Neighborhoods. *JAMA Netw Open*. Published January 09, 2026.
doi:10.1001/jamanetworkopen.2025.51212

Data
Data available: No

Additional Information
Explanation for why data not available: The data that support the findings of this study are available from the California Cancer Registry. Restrictions apply to the availability of these data, which were used under license for this study. Data are available from the California Cancer Registry via application at http://www.ccrcal.org/.
